# Prevalence of diabetic peripheral neuropathy in Iran: a systematic review and meta-analysis

**DOI:** 10.1186/s40200-014-0097-y

**Published:** 2014-10-15

**Authors:** Sahar Sobhani, Hamid Asayesh, Farshad Sharifi, Shirin Djalalinia, Hamid Reza Baradaran, Seyed Masoud Arzaghi, Morteza Mansourian, Aziz Rezapoor, Hossein Ansari, Mohammad Parvaresh Masoud, Mostafa Qorbani

**Affiliations:** Non-Communicable Diseases Research Center, Endocrinology and Metabolism Population Sciences Institute, Tehran University of Medical Sciences, Tehran, Iran; Department of Medical Emergencies, Qom University of Medical Sciences, Qom, Iran; Elderly Health Research Center, Endocrinology and Metabolism Population Science Institute, Tehran University of Medical Sciences, Tehran, Iran; Endocrinology and Metabolism Research Center, Endocrinology and Metabolism Research Institute, Tehran University of Medical Sciences, Tehran, Iran; Development of Research and Technology Center, Deputy of Research and Technology, Ministry of Health and Medical Education, Tehran, Iran; Endocrine Research Center, Institute of Endocrinology and Metabolism, Iran University of Medical Sciences, Tehran, Iran; Department of Public Health, Ilam University of Medical Sciences, Ilam, Iran; Hospital Management Research Center, Department of Health Economic, School of Health Management and Information Sciences, Iran University of Medical Sciences, Tehran, Iran; Health Promotion Research Center, Zahedan University of Medical Sciences, Zahedan, Iran; Department of Community Medicine, School of Medicine, Alborz University of Medical Sciences, Karaj, Iran

**Keywords:** Diabetic peripheral neuropathy, Diabetes mellitus, Systematic review, Meta-analysis

## Abstract

Diabetic peripheral neuropathy (DPN) is an important microvascular complication of diabetes mellitus (DM). It is a major contributor to foot ulceration and lower limb amputation in persons with DM and have also a significant negative effect on patient's quality of life. This meta-analysis reviews prevalence of DPN among patients with type 1 and 2 DM in Iran. Using PubMed and NLM Gateway (for MEDLINE), Institute of Scientific Information (ISI), and SCOPUS as the main international electronic data sources, and Iranmedex, Irandoc, and Scientific Information Database (SID), as the main domestic databases with systematic search capability, we systematically searched surveys, papers, and reports on the prevalence of DPN (between January 1991 to February 2013). Heterogeneity of reported prevalence’s between studies was assessed by the Chi-square-based Q test and due to heterogeneity; overall prevalence of DPN was estimated using random-effect meta-analysis model. We found 304 records; from them a total of 21 studies comprising 5540 diabetic patients were included. The prevalence of diabetic neuropathy (reported) from 16% to 87%. In overall the prevalence of DPN estimated 53% (95% CI: 41-65) by using random-effect. This study show that the prevalence of DPN seems very high among the population with diabetes in Iran and more than half of the patients with DM has any type of diabetic neuropathy.

## Introduction

One of the major complications of diabetes mellitus (DM) is represented by the diabetic peripheral neuropathy (DPN). Neuropathy is the most common complication and greatest source of morbidity and mortality in diabetes patients. It is estimated that the prevalence of peripheral polyneuropathy in diabetes patients is approximately 25-50% in developing countries [1,2]. A DPN account for more hospital admissions than all other diabetic complications combined and is responsible for 50 – 75% of non-traumatic amputations [2,3]. Painful DPN is associated with a high degree of functional impairment, impairment in health-related quality of life and activities of daily living [4,5]. In the literature, the prevalence of Painful DPN ranges from 10% to 20% of patients with diabetes and from 40% to 50% of those with diabetic neuropathies [6]. Painful DPN reportedly results in significantly higher healthcare costs when compared with age and sex-matched diabetic patients without DPN [6,7].

Considering the priority of problem and its increasing co-morbid complication, there is an undeniable need to prepare primary data for more awareness of stakeholders and better policy recommendations [8]. To address this issue, we should provide comprehensive scientific evidence that support policy actions, programs monitoring, and interventions evaluation [9].

This study aimed to assess the prevalence of DPN in Iran (between January 1991 to February 2013) by conducting an up-to-date comprehensive systematic review and meta-analytic comparison of all available studies.

## Methods

### Search strategy

The relevant empirical literature was identified by searching several electronic databases: Main domestic databases; Iran-Medex, Scientific Information Database (SID), Irandoc, and also in international databases; PubMed and NLM Gateway (for MEDLINE), Institute of Scientific Information (ISI), and SCOPUS, between January 1991 to February 2013.

The search was performed by cross-referencing the words “Diabetic Neuropathies” OR “Diabetic Neuropathy” OR “Diabetic Foot” OR “Diabetic Polyneuropathy” OR “Diabetic Polyneuropathies” OR “Diabetic Neuralgia” OR “Peripheral Nervous System Diseases and Iran” OR “I.R. Iran” OR “I R Iran” OR “Persian”.

All Iranian scientific journals that are not listed in the domestic electronic databases, governmental reports, projects reports, conferences and reference lists, were also reviewed by hand searching.

#### Definition

Diabetic neuropathy is a nerve disorder caused by diabetes mellitus. Diabetic neuropathy may be diffuse, affecting several parts of the body, or focal, affecting a specific nerve and part of the body [1,10].

The typical DPN is a chronic, symmetrical, length-dependent sensorimotor polyneuropathy (DSPN) and is thought to be the most common variety [10]. It develops on (or with) a background of long-standing hyperglycemia, associated metabolic derangements (increased polyol flux, accumulation of advanced glycation end products, oxidative stress, and lipid alterations among other metabolic abnormalities) and cardiovascular risk factors [10,11].

The quality assessment of eligible papers has been followed independently by two research experts and probable discrepancy between them resolved based on third expert opinion. Using Cohen’s kappa statistic, agreement of them in quality assessment was 0.92.

#### Inclusion and exclusion criteria

We included all available hospital-based or clinic-based studies. We excluded article with duplicate citation.

#### Data extraction

Data were collected according to a standard protocol independently by two authors. Disagreement was resolved by discussion between them. In cases they could not reach a consensus, a third author was consulted. The extracted information from literature included the name of the first author, the year of publication, the study region, total sample size, age and sex groups, diabetes type, the duration of diabetes, reported prevalence and its 95% confidence interval.

### Statistical analysis

The reported prevalence is presented as percent and 95% confidence interval (CI). Heterogeneity of reported prevalence’s between studies was assessed by the Chi-square-based Q test and I square statistics. The result of Q test was regarded to be statistically significant at P < 0.1. Due to sever heterogeneity among studies regarding reported prevalence of DPN in Iran, overall prevalence was estimated using random-effect meta-analysis model (using the Der-Simonian and Laird method). Forest plot also was used to present result of meta-analysis schematically. The analyses were conducted with STATA software, version 11.0.

## Results

**Figure 1:**
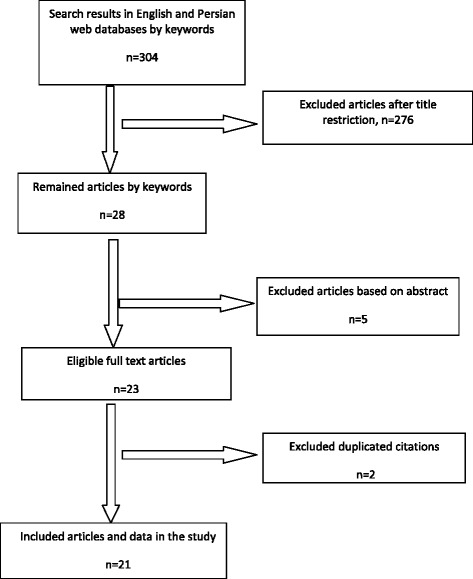
**Flow diagram of the study selection process.**

The flow diagram of the study selection process is shown in the Figure 1. The search yielded 304 publications that were related to inclusion criteria. According to titles, 276 publications were excluded as clearly ineligible, leaving 28 for further review. Where possible, we obtained copies of the full published version of each study, which were then carefully assessed against inclusion/exclusion criteria. After reading abstracts, five publications were excluded at this stage, leaving 23 publications that were provisionally eligible. Finally after exclusion of two duplicated publications, 21 studies were fully eligible for inclusion in this meta-analysis.

Of these 21 studies, 15 had been reported DPN prevalence only as totally without considering gender and diabetes types, while two studies had data on DPN prevalence among males and females and also four papers had distinguished DPN prevalence in both diabetes type 1 and 2. Two studies assessed the prevalence of DPN only in patients with type 2 diabetes and also one study had been included type 1 diabetes. Eighteen studies had samples from both type of diabetes. The extracted data from these studies are shown separately in Tables 1 and 2.

**Table 1:** **The Characteristic of extracted articles for diabetic peripheral neuropathy in Iran**

**Number**	**Reference**	**Location**	**Year**	**Mean age (SD)**	**Sample size (n)**	**Duration (yr)**	**Consideration**
1	Kiani et al. (2013) [10]	Hamadan	2011	53.26 (14.8)	Type 1: 79	9.5 (7.2)	
					Type 2: 521	Type 2	
						9.2 (7.4)	
2	Hasani et al. (2013) [12]	Isfahan	2008-2009	11.9 (3.3)	T: 146	3.8 (2.9)	Only type 1 diabetes
					M: 62		
					F: 84		
3	Bostani et al. (2011) [13]	Mashhad	2008-2009	53.2 (1.8)	T: 110	0-30	
4	Talaee et al. (2011) [14]	Kashan	2008-2010	-	T: 352	-	
5	Tabatabei Malazi et al. (2011) [15]	Tehran	2004	53 (12)	T: 124	10 (8)	
6	Cheraghi et al. (2010) [16]	Shadegan	2009	≥30	T: 521	-	
7	Afkhami-Ardekani et al. (2009) [17]	Yazd	2006-2007	55.9 (10.0)	T: 1000	11.7 (6.8)	Only type 2 diabetes
					M: 457		
					F: 543		
8	Sadeghieh Ahari et al. (2009) [18]	Ardebil	2003	52.5 (11.3)	T: 110	8 (6.2)	Only type 2 diabetes
9	Ghorbani et al. (2008) [19]	Isfahan	2003	40 (-) Range: 15-65	T: 446	-	
					M: 131		
					F: 315		
10	Abbasian et al. (2008) [20]	Shahrod	-	50.2 (15.2)	T: 340*	5.5 (-)	
12	Baghani Moghdam et al. (2007) [21]	Yazd	-	60% ≥50 yr	T: 120	-	
				40% < 50 yr			
15	Ghavami et al. (2007) [22]	Oromieh	2002-2003	Renge: 40-65	T: 30	-	
					M: 9		
					F: 21		
11	Khazai et al. (2006) [23]	Mashhad	2002	52.2 (-)	T: 200	-	
					M: 134		
					F: 66		
13	Janghorbani et al. (2006) [24]	Isfahan	2000-2003	52.7 (9.9)	T: 810	8.2 (6.8)	
					M: 289		
					F: 521		
					Type 1: -		
					Type 2: 810		
14	Yazdanpanah et al. (2006) [25]	Dena Kohkeloieh	2003	60% ≥60 yr	T: 40	-	
					M: 12		
					F: 28		
					Type 1: -		
					Type 2: 40		
16	Madani et al. (2006) [26]	Tehran	2003	59.8 (10.1)	T: 68	10.3 (6.7)	
17	Ranjbar Omrani et al. (2002) [27]	Shiraz	Retrospective studying recent 12 years 1990-2002	Type 1	T: 392	11.4 (6.7)	
				47.5 (10.4)	Type 1: 92		
				Type 2	Type 2: 300		
				20.4 (12.8)			
18	Sarshar et al. (2003) [28]	Gonabad	2001	Type 1	T: 162	-	
				52 (-)			
				Type 2			
				23 (-)			
19	Tegha et al. (2002) [29]	Tehran	1995-1996	-	T: 188	-	
20	Rezvani et al. (2001) [30]	Birjand	1999-2000	≥30	T: 70	-	
21	Habibi Moeini et al. (1999) [31]	Eslamshahr	-	47 (10)	T: 51	-	

*0.3% other type of DM.

**Table 2:** **The prevalence and 95% CI of diabetic peripheral neuropathy in extracted studies in Iran**

**Number**	**Reference**	**Prevalence (%)**	**95% CI**	**Consideration**
1	Kiani et al. (2013) [10]	Total: 45.7	Total: 41.6, 49.7	
		Type 1: 21.5	Type 1: 13.1, 32.2	
		Type 2: 49.3	Type 2: 44.9, 53.7	
2	Hasani et al. (2013) [12]	T: 27.4	T: 20.3, 35.4	Only type 1 diabetes
3	Bostani et al. (2011) [13]	T: 87.3	T: 84.8, 89.4	
4	Talaee et al. (2011) [14]	T: 15.6	T: 14.0, 17.2	
5	Tabatabei Malazi et al. (2011) [15]	T: 38	T: 34.0, 42.0	
6	Cheraghi et al. (2010) [16]	T: 81.9	T: 80.9, 82.8	
7	Afkhami-Ardekani et al. (2009) [17]	T: 51.9	T: 48.7, 55.0	Only type 2 diabetes
		M: 21	M: 19.0, 23.0	
		F: 51.6	F: 47.2, 55.8	
8	Sadeghieh Ahari et al. (2009) [18]	T: 29.1	25.3, 33.1	Only type 2 diabetes
9	Ghorbani et al. (2008) [19]	T: 77.4	T: 76.0, 79.0	
		M: 29.4	M: 21.4, 37.5	
		F: 70.6	F: 65.1, 75.4	
10	Abbasian et al. (2008) [20]	T: 77.3	T: 74.8, 79.4	0.3% other type of DM
12	Baghani Moghdam et al. (2007) [21]	T: 87	T: 85.0, 89.0	
15	Ghavami et al. (2007) [22]	T: 76.5	T: 69.9, 83.1	
11	Khazai et al. (2006) [23]	T: 41.6	T: 38.0, 45.2	
		M: 21	M: 14.4, 28.7	
		F: 51.6	F: 38.9, 64.0	
13	Janghorbani et al. (2006) [24]	T: 75.1	T: 74.1, 76.1	
		M: 35.5	M: 29.2, 40.4	
		F: 64.5	F: 60.2, 68.6	
14	Yazdanpanah et al. (2006) [25]	T: 52.2	T: 44.2, 60.2	
16	Madani et al. (2006) [26]	T: 63.2	58.0-68.4	
17	Ranjbar Omrani et al. (2002) [27]	T: 68.8	T: 67.0, 71.0	
		Type1: 67	Type1: 55.6, 75.8	
		Type2: 69	Type2: 63.4, 74.2	
18	Sarshar et al. (2003) [28]	T: 40.7	T: 37.1, 44.2	
19	Tegha et al. (2002) [29]	T: 35.1	T: 32.1, 38.1	
20	Rezvani et al. (2001) [30]	T: 44.3	T: 38.8, 50.4	
21	Habibi Moeini et al. (1999) [31]	T: 33.3	T: 26.8, 39.4	

The Bostani et al. study in Mashhad found highest prevalence of DPN, with a prevalence of 87.3% [13] and the lowest DPN prevalence (15.6%) was found in Talaee et al. study conducted in Kashan [14]. The highest and lowest DPN prevalence in woman was 70.6% and 51.6% respectively. The study in Isfahan found the highest DPN prevalence of 35.5% in men and the lowest prevalence of DPN (21%) in men was reported by Afkhami-Ardekani et al. and Khazai et al. study [17,23].

Only two studies had been reported DPN prevalence in both type 1 and 2 of diabetes. A study in Hamedan found prevalence of DPN 21.5% in type 1 diabetes and 49.3% in type 2 diabetes [10]. The other study in Shiraz had been estimated DPN prevalence in both type 1 and 2 of diabetes 67% and 69% respectively [27]. One study was conducted only among patients with type 1 of diabetes and has been reported DPN overall prevalence as 27.4% [12].

**Figure 2:**
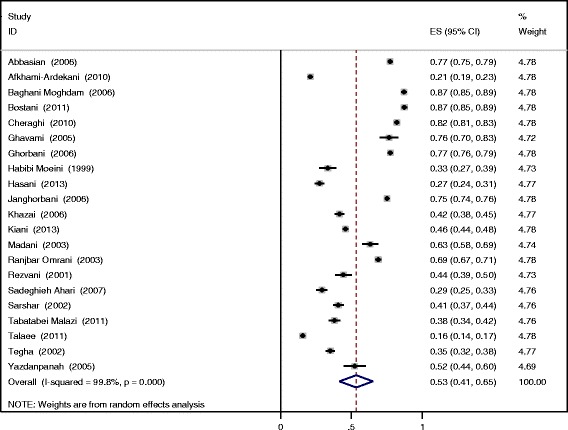
**Forest plot of DPN prevalence in Iran using random-effect model.**

The results of heterogeneity test show sever heterogeneity among reported prevalence (I^2^: 99.8%, p-value < 0.01) and due to heterogeneity, random-effect meta-analysis was performed. Based on extracted article and random-effect meta-analysis; overall DPN estimated prevalence was 53% (95% CI: 41-65). Figure 2 present the forest plot of eligible articles for estimating DPN prevalence in Iran.

## Discussion

This is an updated systematic review of reported prevalence of diabetic neuropathy among the Iranian population. In our study finally 21 studies were eligible for inclusion and data about them from overall of Iran considered for meta-analysis. We found a few studies about neuropathy among patients with diabetes mellitus type I and it is not possible to conduct a meta-analysis of data of this group.

The prevalence of DPN in this meta-analysis estimated 53% in patients with type 2 diabetes. In comparison with other similar studies in developing countries, this estimated prevalence of DNP in Iran seems significant higher than those reports [1,2].

However the prevalence of DNP in our meta-analysis was point estimated but it varied from 16% to 87%, this variation could be justified by the different diagnostic criteria of diabetic neuropathy, the age of the participants in each study, duration and severity of diabetes in their participants as well as the response rate of the study population.

This frequency is higher that the studies which reported the prevalence of peripheral DPN in developing countries [1,2].

A multi-center hospital-clinic based study in the UK the overall prevalence of DPN was reported 32.1% (95%CI: 30.6-33.6%) in diabetes type II and 22.7% (95% CI: 21.0-24.4%) in DM type I. They found that with increase of age and duration of DM the prevalence of DPN increases and the prevalence of peripheral neuropathy in ≥60 years age group is higher than 50% (100) [32].

In another large community based study in Rochester was demonstrated that 59% of patients with diabetes type II and 66% of patients with diabetes type I had some form of diabetes neuropathy [33]. In another multi-center study in Italy only 16.5% of patients with diabetes mellitus were not neuropathic 19.4% neuropathy was borderline and other diabetic participants had some degrees of neuropathy [34].

On the other hand, the studies, which were included in this review, had very diversified in the prevalence of diabetic neuropathy that they reported from 16% in Tabatabei Malazi et al. to 87% in Baghani Moghdam et al. This diversity could depend upon the diagnostic criteria of diabetic neuropathy, the age of the participants in each study, duration and severity of diabetes in their participants as well as the response rate of the study population. Hyperglycemia due to diabetes mellitus increases release of cytokines and activates of protein C kinase and other oxidative stress [35]. The interesting notice is that these processes are time dependent and exacerbate with worse glycemic control [36,37].

According to our knowledge this is the second systematic review of prevalence of diabetic neuropathy in Iran which updates results of pervious study [38]. We enrolled all studies, which had reported diabetic neuropathy in all age groups up to 2013 in Iran.

### Limitations

The first is that we could not report the prevalence of diabetic neuropathy based on its severity. Other one was that we could not extract enough data for estimating of aged adjusted prevalence of the diabetic neuropathy and difference in age of studies population may be one of the sources of heterogeneity between studies included in study and discrepancy between the findings of this study with other studies reported the prevalence of neuropathy. The studies, which were also considered for analysis, might not use the similar criteria for diagnosis of diabetic neuropathy.

## Conclusion

In conclusions it seems that the prevalence of neuropathy is very high among the population with diabetes in Iran and more than half of the patients with DM has any type of diabetic neuropathy.

The present findings could provide practical information on DPN in Iran. These could be useful for better health policy and more detailed studies in this field. The presented results also could be used for future complementary analyses in sub-national, national or even global levels.

## Abbreviations

DPN: Diabetic peripheral neuropathy
DSNP: Length-dependent sensorimotor polyneuropathy
DM: Diabetes mellitus
